# Competition between Starter Cultures and Wild Microbial Population in Sausage Fermentation: A Case Study Regarding a Typical Italian Salami (*Ventricina*)

**DOI:** 10.3390/foods10092138

**Published:** 2021-09-10

**Authors:** Chiara Montanari, Federica Barbieri, Fausto Gardini, Giulia Tabanelli

**Affiliations:** 1 Interdepartmental Centre for Industrial Agri-Food Research, University of Bologna, 47521 Cesena, Italy; chiara.montanari8@unibo.it (C.M.); fausto.gardini@unibo.it (F.G.); 2 Department of Agricultural and Food Sciences, University of Bologna, 47521 Cesena, Italy; federica.barbieri16@unibo.it; 3 Department of Agricultural and Food Sciences, University of Bologna, 40127 Bologna, Italy

**Keywords:** traditional fermented sausages, starter cultures, competition, biogenic amine, off-odour, isovaleric acid

## Abstract

The work reports a case study describing how the competition wild microflora vs. starter cultures affects the final product characteristics. This study regards an industrial lot of *Ventricina*, an Italian long-ripened traditional fermented sausages, produced using starter cultures. After ripening, some relevant organoleptic defects (off-odour, crust formation) were observed. Therefore, analyses were carried out in the inner and outer sausage section to explain this phenomenon. Microbiological analyses indicated a high meat batter contamination and metagenomic analyses evidenced the inability of LAB starter cultures to lead the fermentation process. The results of this not controlled fermentation were the accumulation of high levels of biogenic amines (including histamine) and the formation of a volatile profile different if compared with similar products. Indeed, the volatilome analysis revealed unusually high amounts of molecules such as isovaleric acid, propanoic acid, 1-propanol, which can be responsible for off-odours. This study demonstrated that starter culture use needs to be modulated in relation to production parameters to avoid safety and organoleptic concerns.

## 1. Introduction

*Ventricina* is a fermented sausage typically produced in some Italian areas comprised between Abruzzo and Molise Regions. Despite the wide variability of local recipes, *Ventricina* is usually obtained by cutting pork lean meat, which represents about 80% of sausage, in rather big cubes (3–4 cm). The remaining fat part is constituted by bacon and thigh fat. In addition to salt and pepper, sometimes powdered sweet pepperoni and fennel flowers/seeds may be added. Traditionally, this sausage is stuffed in pork stomach for ripening, explaining the etymology of the Italian name (*ventre* = stomach). At an industrial level, it is stuffed in natural (pig bladder or veal caecum) or synthetic casings and ripened at low temperature for at least 100 days. The weight of the final product ranges from 1 to 2.5 kg. After the first 50 days of ripening, the external part is covered with pork lard to avoid excessive water losses [1,2].

In the absence of specific constraints due to protective marks (PDO, PGI), *Ventricina* may be produced everywhere, and many fermented meat industries located outside the traditional Regions of Italy produce this kind of sausage.

Likewise, in many other traditional salamis, the practices for *Ventricina* production have been modified applying industrial processes able to guarantee the safety and the standardization of the product, especially in the framework of the drastic reduction of salt following the request of markets, nutritionists, and consumers. This approach firstly requires the addition of starter cultures, sugars, and a rigorous control of temperature and humidity during fermentation and ripening [3].

The use of starter cultures (lactic acid bacteria, staphylococci, and moulds) became in the last decades a widespread operation in the fermented sausage industry. They are usually composed of lactic acid bacteria (lactobacilli and pediococci) and staphylococci, whose growth in the meat mixture is essential to inhibit the proliferation of pathogens, to impart the desired flavour, to control the colour formation, and to favour a correct water loss [4].

Starter cultures have to establish environmental supremacy with the microorganisms present in the raw material in order to obtain a product with the desired characteristics and to guarantee the safety issues of fermented sausages. However, this practice is still applied as an empirical operation and industrial operators sometimes perceive it as the adjunct of an additive, having little attention to this crucial production step and to the essential role carried out by this viable substrate. This role is dependent on many process factors (NaCl and nitrate/nitrite concentration, temperature) and raw material characteristics, first of all, the microbial quality of the meat, i.e., the qualitative and quantitative presence of wild microorganisms. The success of the competition depends on the first instance of the numerical ratio between starter culture added and microorganisms present. The initial contamination of meat in industrial production can be extremely variable: usually counts comprised between 3.2 and 5.3 log CFU/g are reported [5]. This variability is the consequence of slaughtering operations and eventual freezing/thawing procedures. Generally, a minimum ratio of 10:1 between starter cultures e natural microflora should be assured to avoid an inadequate activity or competitiveness of starter cultures towards the wild microbiota, which may cause several problems in the fermented sausages, raising both organoleptic and safety concerns.

This work is aimed to describe the defects that occurred in the industrial production of *Ventricina* as the results of the failed competition between the starter cultures added during manufacturing and the wild microorganisms present in the raw materials. For this reason, a metagenomic analysis on the microbial communities of the final product was performed, taking into consideration two different sections of the sausages, i.e., the internal part and the external part. In addition, the aroma profile through SPME-GC-MS and the biogenic amine content were determined.

## 2. Materials and Methods

### 2.1. Sausage Manufacture

The samples of *Ventricina* used in this study were industrially produced in an Italian company using frozen pork shoulder (90%) and lard cubes. The meat (600 kg) was minced (3–4 cm) and added with dextrose (0.1%), sodium chloride (2.6%), spices (black pepper, red pepper, fennel), nitrates and nitrites, commercial starter cultures (*Latilactobacillus sakei*, *Pediococcus pentosaceus*, *Staphylococcus xylosus,* and *Staphylococcus carnosus* (Biostart SPL-600, Probiotec, Villanova sull’Arda, Italy)). The meat mixture was then stuffed in a synthetic collagen casing (diameter 11 cm and initial weight approx. 4 kg) and transferred in the fermentation/ripening chamber under controlled conditions of temperature and relative humidity (RU): 3 days at 23 °C, RU 80–90%; 4 days at 17 °C, RU 60–90%; then 13 °C, RU 75–80% until the end of ripening (100 days). Samples were collected during fermentation and ripening to monitor pH, water activity, and weight loss. The final products were analysed to assess microbiota (with both culture-dependent and independent methods), biogenic amine content, and volatile profiles. For these analyses, two different parts of the sausages were considered: the outer section (3 cm of products immediately below the casing) and the inner one (center of the sausages). For each sampling, three different sausages were collected, each one analysed in triplicate, both for the inner and outer parts.

### 2.2. Weight Loss, a_w_ and pH

Samples were weighed during the whole process to calculate the mean weight loss (%) with respect to the initial one; a_w_ was measured in triplicate with an Aqualab CX3-TE (Labo-Scientific, Parma, Italy). The pH of fermented sausages during fermentation and ripening was determined in the inner and outer part of the product using a pH-meter Basic 20 (Crison Instruments, Barcelona, Spain).

### 2.3. Microbial Counts

After aseptically removing the casing, approx. 10 g of sausage were 10-fold diluted with 0.9% (*w*/*v*) NaCl and homogenized in a Lab Blender Stomacher (Seward Medical, London, UK) for 2 min. Decimal dilutions were performed and plated onto selective media. Counts of lactic acid bacteria (LAB) were carried out by plating appropriate dilutions on MRS agar incubated at 30 °C for 48 h in anaerobic conditions. Staphylococci and enterococci were counted by surface-plating on Baird-Parker (added with egg yolk tellurite emulsion), and Slanetz and Bartley medium incubated at 30 °C for 48 h and 44 °C for 24 h, respectively. *Enterobacteriaceae* and total coliforms were enumerated by pour plating in Violet Red Bile Glucose Agar and Violet Red Bile Lactose Agar, respectively, and then incubating the plates at 37 °C for 24 h. The total mesophilic count was determined on Plate Count Agar (28 °C for 48 h), while *Pseudomonadaceae* counts were performed on Pseudomonas Agar Base, supplemented with Pseudomonas CFC Supplement and incubated at 30 °C for 48 h. All media and supplements were purchased from Oxoid, Basingstoke (UK).

### 2.4. DNA Extraction and Sequencing

Total genomic DNA was directly extracted from 10 g of frozen *Ventricina*. The samples were dissolved in 90 mL of physiological solution (0.9% NaCl) and homogenized in a stomacher for 4 min at 430 beats per minute. After decanting, 1 mL of the supernatant was collected and subjected to enzymatic treatment towards bacteria (lysozyme) and yeasts (lyticase) at 37 °C for 1 h, followed by alkaline lysis with the addition of NaOH and SDS at a final concentration of 0.1 N and 1%, respectively. The extracted DNA was purified by chloroform:isoamyl alcohol 24:1 treatment and precipitated in 0.54 volumes of isopropanol. Finally, the purified DNA was resuspended in water and quantified using Qubit 4 Fluorimeter (ThermoFisher Scientific, Waltham, MA, USA). The concentration of the DNA samples was normalized, and the sequencing was carried out through the Illumina MiSeq platform which generated 300 bp pair-end sequencing reads. The library for Illumina sequencing was generated from V3-V4 variable regions of ribosomal 16S rRNA in order to characterize the bacterial population of the samples.

### 2.5. Bioinformatic Analysis

FASTQ sequence files from Illumina reads were generated using bcl2fastq2 version 2.18. Initial quality assessment was based on data passing the Illumina Chastity filtering. Subsequently, reads containing PhiX control signal were removed using an in-house filtering protocol. In addition, reads containing (partial) adapters were clipped (up to a minimum read length of 50 bp). A final quality assessment was performed on the remaining reads using the FASTQC quality control tool version 0.11.5. The FASTQ sequences obtained were analysed using DADA2 version 1.8 by R 3.5.1 environment. DADA2 implements a new quality-aware model of Illumina amplicon errors without constructing OTUs [6]. DADA2 was run as described in https://benjjneb.github.io/dada2/tutorial.html (accessed on 6 September 2021) applying the following parameters: trimLeft equal to 30 and truncLen option set to 270 and 200 for the forward and reverse fastq files, respectively. The taxonomic assignment was performed comparing the amplicon sequence variant (ASV) predicted from DADA2 against the SILVA database (version 128, https://www.arb-silva.de/documentation/release-128/; accessed on 6 September 2021). ASVs belonging to taxa classified as external samples [7] contaminations were not included in the composition analysis for the microbial population.

### 2.6. Organic Acid and Glucose Quantification

The extraction of organic acids was performed according to Montanari et al. [8]: 10 g of each sample were added with 40 mL of 0.05 mM sulfuric solution, homogenized for 10 min by an Omni Mixer Homogenizer (Omni International, Warrengton, VA, USA) and filtered through a 0.22 μm filter. The extract analysis was performed by using an HPLC (PU-2089 Intelligent HPLC quaternary pump, UV–VIS multi-wavelength detector UV 2070 Plus, Jasco Corp., Tokyo, Japan) and a manual Rheodyne injector with a 20 μL loop (Rheodyne, Rohnert Park, CA, USA), equipped with a Bio-Rad Aminex (Bio-Rad Laboratories, Hertfordshire, UK) HPX-87H column (300 × 7.8 mm). The following conditions were used: mobile phase, 0.005 M sulfuric acid; flow rate, 0.60 mL/min; temperature, 65 °C; the UV detector was set at 210 nm. Chromatographic peaks were identified by comparing retention times with those of standards (Sigma-Aldrich, St. Louis, MO, USA) and quantification was carried out by using the external standard method.

For the extraction of glucose, 3 g of sample were dissolved in 15 mL of water and defatted by adding 30 mL of n-hexane followed by stirring for 30 min at room temperature (22 °C). The samples were centrifuged at 4000 rpm for 10 min and treated as reported by Tabanelli et al. [9]. A HPLC apparatus (Agilent Technologies 1200 series) equipped with 2 binary pumps, an evaporative light scattering detector (ELSD), and a Luna NH2 250 × 4.6 mm, 5 μm column (Phenomenex Inc., Torrance, CA, USA.) was used. Glucose was eluted with a water: acetonitrile 30:70 (*v*/*v*) solution as mobile phase (flow rate 1.0 mL/min) in isocratic mode.

### 2.7. Biogenic Amine Content

For the detection of biogenic amine (BA) content, samples were subjected to an extraction with trichloroacetic acid followed by derivatization with dansyl chloride, as described by Pasini et al. [10]. The biogenic amines content was analysed using a HPLC Agilent 1260 Infinity (Agilent Technologies Inc., Santa Clara, CA, USA) with a UV detector (G1314F VWD 1260) at 254 nm. The amounts of amines were expressed as mg/L by reference to a calibration curve obtained with aqueous biogenic amine standards derivatized as described for the samples.

### 2.8. NaCl Concentration

The determination of salt concentration was carried out using Near Infrared Transmission with FoodScan instrument for non-homogeneous products, such as salami (Foss, Hilleroed, Denmark).

### 2.9. Aroma Profile Analysis

Volatile organic compounds of samples were analysed with gas chromatography-mass spectrometry coupled with solid-phase microextraction (SPME-GC-MS), using an Agilent Hewlett–Packard 6890 GC gas-chromatograph and a 5970 MSD MS detector (Hewlett–Packard, Geneva, Switzerland) equipped with a Varian (50 m × 0.32 mm × 1.2 μm) fused silica capillary column. Samples (3 g) were placed in 10 mL sterilized vials, added with a known amount of 4 methyl- 2-pentanol (Sigma-Aldrich, Steinheim, Germany) as internal standard, and sealed by PTFE/silicon septa. The samples were heated for 10 min at 45 °C and then a fused silica SPME fiber covered with 85 μm Carboxen/Polydimethylsiloxane (CAR/PDMS) (Supelco, Steinheim, Germany) was introduced into the headspace for 40 min. Adsorbed molecules were desorbed in the gas-chromatograph for 10 min. The conditions were the same reported by Montanari et al. [8]. Volatile peak identification was carried out by computer matching of mass spectral data with those of compounds contained in the libraries NIST 2005 and 2011. Data reported are the means of three different sausages.

### 2.10. Statistical Analysis

Three independent different sausages were investigated with each analysed in triplicate. The data were statistically analysed using the Mann-Whitney test to determine differences among different conditions (*p* < 0.05). All statistical analyses were carried out by using Statistica 8.1 (StatSoft Italy srl, Vigonza, Italy).

## 3. Results and Discussion

### 3.1. Analyses of the Meat Mixture and Control of Ripening Parameters (pH, Weight Losses and a_w_)

The viability of the starter cultures was assessed before use and the results were 9.80 (±0.32) and 10.00 (±0.29) log CFU/g of dried culture for LAB and coagulase-negative staphylococci (CNS), respectively. These initial concentrations, combined with the dilution used during the meat mixture preparation, determined a LAB inoculum of about 5.5 log CFU/g and a CNS inoculum of about 5.7 log CFU/g. The meat mixture used for sausage preparation was analysed before the addition of starter cultures and the results evidenced relevant microbial contamination. In particular, the LAB count was 5.78 (±0.10) log CFU/g, CNS 5.85 (±0.12) log CFU/g, total coliforms 2.90 (±0.10) log CFU/g and *Enterobacteriaceae* 2.45 (±0.17) log CFU/g. In addition, pH (5.70 ± 0.02), NaCl content (2.64% ± 0.01) and glucose concentration (0.49 ± 0.03 g/kg) of the meat mixture were determined.

**Figure 1:**
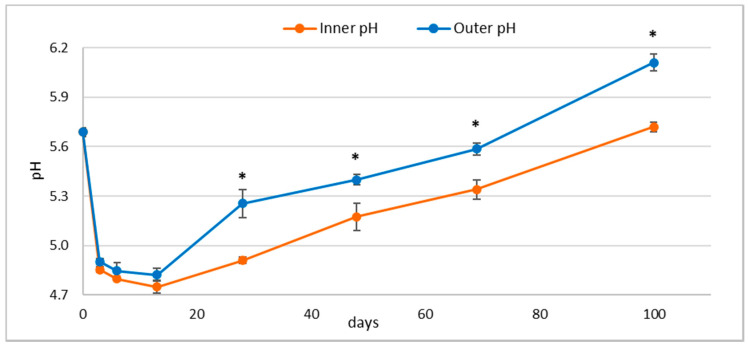
Evolution of pH during the 100 days ripening period in the inner and in the outer section of *Ventricina* samples. Standard error bars are showed. Sampling times with an asterisk were characterized by significant differences between samples according to Mann-Whitney test.

During drying and ripening, pH, weight losses, and a_w_ were monitored. Regarding pH, measurements were carried out in the outer and inner sections. The results (Figure 1) showed that the initial pH value of 5.70 decreased to 4.75 at the end of fermentation (approx. 13 days). After this phase, it constantly increased to reach, after 100 days, a value close to the initial one in the inner part (5.70). A different behaviour characterized the outer part in which the pH reduction was slightly lower, followed by a rapid increase (5.28 after 28 days). During the long ripening, pH continued to increase. At the end of ripening the pH of the outer part was, as expected, higher than the inner part.

These differences, related to the respiration of lactate, can be explained by the activity of mould grown on the casings, and by the activity of staphylococci which take advantage of the higher oxygen concentration immediately below casings.

**Figure 2:**
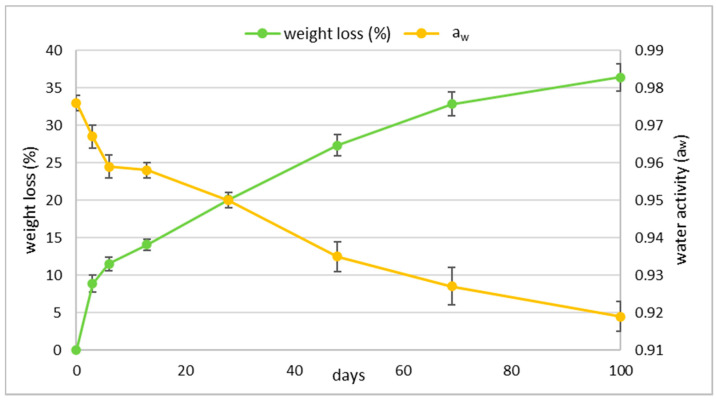
Evolution of weight loss and water activity (a_w_) during the 100 days ripening period of *Ventricina* samples. Standard error bars are showed.

The weight losses and a_w_ during fermentation and ripening are reported in Figure 2. The sausage weight, after a rapid diminution of about 12% after six days slowed its decrease reaching a reduction of 36.4% at the end of ripening. The a_w_ consequently decreased reaching a value of 0.919 after 100 days. However, it is interesting to note that between 6 and 13 days it remained constant at rather high values (0.958) despite the diminution of weight at the same time.

### 3.2. Microbiological Analyses and Organic Acid Concentrations of the Ripened Sausages

At the end of ripening an olfactive defect in the sausages was present, consisting of off-odours which can be defined as sweaty, pungent, putrefactive. In addition, sausages were characterized by the formation of a crust on the outer side. For these reasons, the microbial population present in the inner and the outer part of the final sausages was analysed (Table 1).

LAB were the dominant group with counts higher than 8 log CFU/g in all the samples, without significant differences in relation to the sausage sections. Conversely, for CNS significant differences were observed and levels higher than 7 log CFU/g were reached only in the outer sections. At the end of ripening both enterobacteria and pseudomonads were below the detection limits (1 log CFU/g), while enterococci were detected in low amounts (less than 2 log CFU/g) only in the inner part of the samples.

In the same table, lactic and acetic acid concentrations are reported. Relevant differences were observed between the sections. Indeed, lactic acid had a concentration higher than 11 g/kg in the inner samples, while in the outer part its concentration was 6.01 g/kg. The consumption of lactate is generally attributed to fungi (moulds and, eventually, yeasts). Nevertheless, also staphylococci, in the absence of sugars, can use lactate as the energy source for growth [11]. Acetic acid presented higher concentrations in the inner section (2.32 g/kg) with respect to the outer one (1.47 g/kg).

### 3.3. Metagenomic Analyses

Metagenomic analysis was carried out to verify the contribution of different microbial populations (including starter cultures) to sausage fermentation and ripening. Also, in this case, the inner and the outer part were analysed separately.

A total of more than 800 amplicon sequence variants (ASVs) were detected. In Table 2 the percentage of the ASVs attributed to species or genera in the different sausage sections is shown. Only species and genera which reached a concentration higher than 0.5% in at least one of the samples are reported. Among the most important genera, *Staphylococcus* spp. was present in extremely different proportions in the inner and outer parts of the sausages (31.34 vs. 84.71% of ASVs, respectively). The relevance of staphylococci in the external part of these sausages can be related to higher oxygen availability. The protocol applied in the present study did not allow the identification at the species level. In any case, it cannot discriminate between added and autochthonous staphylococci, which were present in the meat batter after casing in similar proportion with respect to starter cultures added. Noteworthy, the prevalence of staphylococci showed by the metagenomic analysis did not confirm the data of plate counting according to which LAB counts were higher (more than 1 log unit) than staphylococci, even in the outer section. On the other hand, this analytical approach gave a picture of the microbial population throughout all the productive processes, rather than information about viable and cultivable cells at a given time (in this case after 100 days of ripening).

Lactobacilli were identified at genus level, according to the revision of the Genus *Lactobacillus* recently proposed [12]. Several differences were observed regarding the inner/outer part of sausages. Firstly, they represented 56.32% and 10.20% of ASVs in the inner part and in the outer part, respectively. Six different Genera were detected: *Latilactobacillus, Lactiplantibacillus, Lentilactobacillus, Loigolactobacillus, Companilactobacillus* and *Lacticaseibacillus*. The quantitative composition of the lactobacilli community demonstrated the inability of the starter cultures to dominate the environment. In fact, *Latilactobacillus* spp. (presumably the *Lat. sakei* strain used as starter culture) represented only the 26.15% of ASVs in the inner part of *Ventricina*, while it accounted only for 1.31% of ASVs in the outer part of the samples. The Genus *Lactiplantibacillus*, which includes the species *Lpb. plantarum* and *Lpb. paraplantarum* often isolated from spontaneously fermented sausages [13], represented the 15.15% of ASV in the internal of sausages, while it was the most representative LAB in the outer part (5.27%).

A relevant proportion of heterofermentative LAB belonging to the genus *Lentilactobacillus* was also observed, i.e., 9.70 and 2.57% in the inner and outer part, respectively. This Genus includes species such as *Len. buchneri*, which are never dominating in sausages fermentation but may be present as contaminants [14]. Lower ASVs percentages were ascribed to other lactobacilli, such as *Companilactobacillus* sp., *Loigolactobacillus* and *Lacticaseibacillus* sp.

Among the other LAB detected, *P. pentosaceus* reached a concentration of 1.15% and 0.08% of ASVs in the inner and the outer part, respectively. This species was added as a starter culture, but it is clear that it did not grow during fermentation and ripening. Other LAB species detected at low concentration were *Tetragenococcus koreensis*, *Lactococcus lactis*, *Weissella* sp., and *Streptococcus* sp. Many of these species have already been described in spontaneously fermented sausages [15,16] and they may derive from the meat of other ingredients used for sausage manufacture.

Other Gram-positive bacteria detected belonged to the *Propionibacteriaceae* (*Cutibacterium* sp.) and *Corynebacteriaceae* (*Corynebacterium accolens*), especially in the inner part of sausages. A relevant presence (1.5% of ASV) of *Actinomyces* sp. was observed only in the outer part of the sausage.

Few Gram-negative bacteria were revealed by the metagenomic analysis. In the inner part of sausage low concentrations of *Bacteroidetes* (*Prevotella conceptionensis* and *Cloacibacterium normanense*) were observed while, among Proteobacteria, *Pseudomonas* sp. prevailed.

### 3.4. Biogenic Amines Determination

The BA amount resulted extremely high in both sausage parts (Table 3). In general, the BA content was higher in the inner part of sausages due to the major activity of decarboxylases in the presence of low oxygen concentration and the response to acid stress determined by the lower pH [17]. Histamine, the most dangerous BA, reached worrying concentrations (223.6 mg/kg) in the internal part of samples. There are no legal limits for BAs in sausages, but a limit of 200 mg/kg of this amine is reported in the European regulation 2073/2005 for some fishery products [18]. The concentration of tyramine was very similar to histamine (about 200 mg/kg in all the samples).

Relevant differences between the outer and inner parts were observed for putrescine and cadaverine. Putrescine, deriving from ornithine decarboxylation, showed concentrations of 351.5 mg/kg inside the sausages, while in the external part the values were 211.1 mg/kg. A similar behaviour characterized cadaverine (produced through lysine decarboxylation), whose concentration was 597.0 mg/kg in the inner section of sausages and 270.8 mg/kg in the outer section. 2-phenylethylamine was accumulated in negligible amounts (lower than 20 mg/kg), similarly in both sections.

The presence of such relevant amounts of BAs is a further indication of the failure of the starter cultures in colonizing the meat environment. In fact, one of the criteria in the selection of microorganisms for food fermentation is the inability to produce these dangerous compounds. In addition, the species *Lat. sakei* is characterized by the lack of amino acid decarboxylase activity, except for some strains able to produce small amounts of putrescine from ornithine deriving from arginine deiminase (ADI) pathway [19,20]. Thus, other bacteria are involved in the decarboxylase activities. Regarding tyramine, it is well known that LAB are the most efficient producers of this BA [21]. Given the enterococci (the most active in tyrosine decarboxylation) counts reported in Table 1, other LAB, among which many lactobacilli (such as *Levilactobacillus brevis*, *Limosilactobacillus fermentum*, *Lpb. plantarum*, *Len. buchneri*, *Lcb. casei*) can be considered responsible for its accumulation [21,22]. Similarly, histamine can be produced by several LAB species, such as *Len. buchneri*, *Lpb. Plantarum,* and *Lcb. casei* [22].

The great production of aliphatic polyamines (cadaverine and putrescine) observed in these sausages is usually associated with a relevant growth of Gram-negative bacteria, and, in particular, of *Enterobacteriaceae* [23]. According to the metagenomic analysis, the presence of ASVs attributable to this family is rather limited. Thus, the production could be again attributed to LAB. In fact, the ability to produce great amounts of cadaverine and putrescine has already been observed in *Lpb. plantarum* [24] and *Levilactobacillus brevis* [25]. Alternatively, an initial growth of Gram-negative bacteria at the beginning of fermentation could be responsible for their accumulation, taking into account that the decarboxylases can be active also outside the cells following bacterial death and cell lysis [26]. In this case, it is possible that the DNA of these cells has been depleted by other bacteria (mainly LAB) during the 100 day ripening period for using nucleosides as carbon source in a limiting nutritional environment, as demonstrated in *Lat. sakei* [27].

### 3.5. Aroma Profile of Sausages

The aroma profile of the final products was analysed using a SPME-GC-MS protocol already described for sausages [8] to investigate the causes of the off-odours that were perceived at the end of ripening. The results are reported in Table 4.

Among aldehydes, benzeneacetaldeyde and benzaldehyde, deriving from phenylalanine metabolism [28], were dominant (approx. 8–10% of the total volatile profile) without significative changes in relation to the section of the sausage considered. These two compounds are often detected in fermented sausages, even if data obtained under the same conditions in other Italian salamis showed a lower proportion with respect to the results obtained in this trial [29]. The presence of aldehydes deriving from lipid oxidation was scarce since only small quantities of nonanal and decanal were detected. Usually, these compounds are present in larger amounts in Mediterranean fermented sausages [8,29]. This scarce presence may be due to the long ripening times, during which further transformations of these molecules can occur, or to the low-fat content of this type of sausage. In addition, the big size of fat cubes used in the recipe can reduce the total fat surface exposed to lipid oxidation.

Alcohols were present with higher content in the outer part of sausages. Usually, ethanol is the main alcohol in Italian salamis [29]. Its presence can be attributed to several pathways and, together with acetic acid, may result from the LAB metabolism of lactate [30]. In this case, 1-propanol showed a higher amount. This compound is usually detected in lower proportions in analogous products [29]. It may be produced by the reduction of the corresponding aldehyde (propanal), which can be formed by oxidative reactions [31]. Alternatively, some LAB (such as *Limosilactobacillus reuteri*) can produce 1-propanol as a result of secondary pathways addressed to NADH regeneration [32]. This molecule is responsible for acid and fermented odour notes [33], and its high concentration may contribute to the odour defect of the ripened product. 2-butanol, resulting from the reduction of 2-butanone, presented a similar behaviour. Benzyl alcohol and phenethyl alcohol are the results of the reduction of benzaldehyde and benzeneacetaldehyde, respectively.

The presence of ketones was low and mainly represented by 2-butanone. High amounts of this ketone were found in Italian sausages with a large diameter characterized by olfactive defects [8]. The presence of methyl ketones in sausages may derive from β-ketoacids produced during β-oxidation carried out by moulds and staphylococci [34,35]. Nevertheless, lactobacilli can produce 2-butanone starting from diacetyl through the action of a diol dehydratase [36]. However, differently from other similar products, the presence of diacetyl or acetoin was never detected in these sausages.

Several esters were detected, even if these compounds accounted for a limited proportion of volatile molecules. They were mainly represented by ethyl or propyl esters and no differences were observed in relation to the sausage section. This high proportion of propyl esters is not common in Italian fermented sausages [29], and it was evidently associated with the unusually high concentration of propionic acid present. Staphylococci are usually responsible for the major esterase activity in sausages [37,38].

Acids represented the main constituents of the volatile profile (58.6% and 49.3% of the total volatiles in the internal and in the outer section, respectively). These percentages are very high if compared with similar products, where these compounds reached a maximum concentration of 25%. Acetic acid was about one-third of the total acids, similar to the levels found in other large diameter Italian sausages [29]. More unusual was the high concentration of other acids, such as propanoic, butanoic, hexanoic, and octanoic acid. All these molecules are characterized by unpleasant flavour notes, described as rancid, sweaty, pungent, cheesy, sour. Propionic acid is the final metabolic product of *Propionibacteriaceae* to which belongs the Genus *Cutibacterium* found in relevant amounts (2.90%) in the internal part of sausages, according to the metagenomic analysis (Table 2).

In addition, a relevant concentration of 3-methylbutanoic acid was detected. According to Smit et al. [39], this acid derives from leucine metabolism, which is firstly deaminated, and then the resulting α-ketoisocaproic acid can be transformed into isovaleric acid following two ways, the first involving the action of a decarboxylase and the second a α-ketoacid dehydrogenase (oxidative decarboxylation). This second way allows the production of ATP and may be advantageous for bacteria in an environment poor in fermentable substrates such as sausages after several weeks of ripening.

The presence of isovaleric acid in fermented sausages is well documented [40]. It is characterized by a high odour activity value and is accumulated mainly at the end of the ripening process [41]. Its contribution to the overall sausage aroma depends on its concentration, which is characterized by mild sweet or fruity notes at low amounts [42], while high concentrations may result in cheese, feet, and dirty socks smell [41]. The possibility to produce isovaleric acid starting from leucine has already been described in *Carnobacterium piscicola* [43], *Staphylococcus carnosus* [44,45], and in aspergilli and penicilli [46]. In particular, the products of leucine metabolism have been considered as metabolic markers for staphylococci activity in fermented meat [47,48].

## 4. Conclusions

The defects presented by the fermented sausages must be attributed in the first instance to a failure in the activity of the selected LAB (*Lat. sakei* and *P. pentosaceus*) added as starter cultures to lead the fermentation. The raw meat was characterized by a high microbial count and the starter cultures were not able to colonize the environment reducing or inhibiting the growth of the wild LAB communities as demonstrated by the metagenomic analysis. The predominance of staphylococci can be the main cause of the off-odour formation. The metagenomic analysis was not able to identify the staphylococcal species and therefore to understand if this defect is due to the strains used as starter cultures (*S. xylosus* and *S. carnosus*) or to other species frequently associated with fermented sausages. In any case, the defects observed (including crust formation and BA accumulation) can be associated with an improper fermentation process, despite the use of starter cultures.

The conclusion of this work is that the addition of a starter culture is necessary but not sufficient to obtain an acceptable quality level. The use of starter culture needs to be modulated in relation to other production parameters. In particular, the initial microbial contamination of meat can affect the ability of the selected bacteria to colonize the environment, bringing to safety (BAs content) and organoleptic (off-odours) concerns.

## Figures and Tables

**Table 1:** Microbiological analyses (log CFU/g) and organic acids concentration (g/kg) of the ripened sausages in the inner and in the outer section. Results are the mean of three independent repetitions. For each microbial group and organic acid significant differences between samples according to Mann-Whitney test are indicated by the presence of an asterisk.

		Inner Section	Outer Section
Microbial groups (log CFU/g)	LAB	8.36 (±0.26)	8.51 (±0.18)
	Staphylococci (CNS) *	6.84 (±0.22)	7.29 (±0.19)
	Total mesophilic count	8.51 (±0.25)	8.50(±0.36)
	*Enterobacteriaceae*	<1	<1
	Enterococci *	1.78 (±0.51)	<1
	*Pseudomonas*	<1	<1
Organic acids (g/kg)	Lactic acid *	11.59 (±1.03)	6.01 (±0.28)
	Acetic acid *	2.32 (±0.16)	1.47 (±0.11)

**Table 2:** Microbial community composition (by 16S rRNA gene V3–V4 region sequencing) of the inner and outer section of the ripened *Ventricina* samples. Only the amplicon sequence variants (ASVs) which reached a concentration higher than 0.5% in at least one of the samples are reported.

Phylum	Class	Order	Family	Identification	Inner Section (%)	Outer Section (%)
Actinobacteria	Actinobacteria	Propionibacteriales	*Propionibacteriaceae*	*Cutibacterium* sp.	2.90	0.09
Actinobacteria	Actinobacteria	Actinomycetales	*Actinomycetaceae*	*Actinomyces* sp.	0.01	1.50
Actinobacteria	Actinobacteria	Corynebacteriales	*Corynebacteriaceae*	*Corynebacterium accolens*	1.35	0.04
Actinobacteria	Actinobacteria	Corynebacteriales	*Corynebacteriaceae*	*Corynebacterium* sp.	0.10	0.62
Firmicutes	Bacilli	Bacillales	*Staphylococcaceae*	*Staphylococcus* spp.	31.34	84.71
Firmicutes	Bacilli	Lactobacillales	*Enterococcaceae*	*Tetragenococcus koreensis*	0.07	1.71
Firmicutes	Bacilli	Lactobacillales	*Lactobacillaceae*	*Lactiplantibacillus* spp.	15.15	5.27
Firmicutes	Bacilli	Lactobacillales	*Lactobacillaceae*	*Latilactobacillus* spp.	26.15	1.31
Firmicutes	Bacilli	Lactobacillales	*Lactobacillaceae*	*Lentilactobacillus* spp.	9.70	2.57
Firmicutes	Bacilli	Lactobacillales	*Lactobacillaceae*	*Loigolactobacillus* spp.	2.47	0.47
Firmicutes	Bacilli	Lactobacillales	*Lactobacillaceae*	*Companilactobacillus* sp.	1.99	0.52
Firmicutes	Bacilli	Lactobacillales	*Lactobacillaceae*	*Lacticaseibacillus* spp.	0.86	0.06
Firmicutes	Bacilli	Lactobacillales	*Lactobacillaceae*	*Pediococcus pentosaceus*	1.15	0.08
Firmicutes	Bacilli	Lactobacillales	*Leuconostocaceae*	*Weissella* sp.	0.19	0.91
Firmicutes	Bacilli	Lactobacillales	*Streptococcaceae*	*Streptococcus* sp.	1.57	0.01
Firmicutes	Bacilli	Lactobacillales	*Streptococcaceae*	*Lactococcus lactis*	0.69	0.05
Proteobacteria	Gammaproteobacteria	Pseudomonadales	*Pseudomonadaceae*	*Pseudomonas* sp.	3.34	0.07

**Table 3:** Biogenic amine content (expressed as mg/kg) of the ripened sausages in the inner and in the outer section. Results are the mean of three independent repetitions. For each compound significant differences between samples according to Mann-Whitney test are indicated by the presence of an asterisk.

Sample	Biogenic Amines (mg/kg)					
	Histamine *	Tyramine *	Putrescine *	Cadaverine *	2-phenyl-Ethylamine *	BA Total Amount *
Inner section	223.6 (±17.2)	226.3 (±15.2)	351.5 (±24.3)	597.0 (±33.2)	15.2 (±4.6)	1413.6 (±21.4)
Outer section	151.2 (±13.7)	181.5 (±6.33)	211.1 (±12.4)	270.8 (±18.4)	10.7 (±5.2)	825.3 (±16.3)

**Table 4:** Aroma profile of sausages detected by SPME-GC-MS in the ripened sausages in the inner and in the outer section. Data are expressed as percentage of the ratio between peak area of each molecule and peak area of the internal standard (4-methyl-2-pentanol). Results are the mean of three independent repetitions and standard deviations are reported in brackets. For each molecule significant differences between samples according to Mann-Whitney test are indicated by the presence of an asterisk.

Molecule	Inner Section (%)	Outer Section (%)
Nonanal	0.50 (±0.17)	0.31 (±0.15)
Decanal	0.03 (±0.04)	0.05 (±0.05)
Benzaldehyde	0.75 (±0.12)	0.97 (±0.23)
Benzeneacetaldehyde	7.27 (±0.83)	9.28 (±1.27)
Total aldehydes	8.55	10.61
Ethyl alcohol *	8.33 (±1.10)	9.79 (±0.78)
2-Butanol *	4.97 (±0.23)	6.28 (±0.41)
1-Propanol *	9.91 (±0.71)	11.51 (±0.23)
Benzyl alcohol	0.62 (±0.42)	0.87 (±0.31)
Phenylethyl alcohol	2.08 (±0.24)	2.24 (±0.33)
Total alcohols	25.91	30.69
2-Butanone *	2.57 (±0.15)	4.63 (±0.66)
3-Penten-2-one, 4-methyl	0.39 (±0.12)	0.66 (±0.31)
2-Nonanone	0.05 (±0.07)	0.18 (±0.15)
Total ketones	3.01	5.47
Ethyl Acetate	0.61 (±0.11)	0.58 (±0.16)
Propanoic acid, ethyl ester	0.40 (±0.08)	0.38 (±0.13)
n-Propyl acetate	0.98 (±0.21)	0.87 (±0.09)
Propanoic acid, propyl ester	0.93 (±0.23)	1.07 (±0.09)
Octanoic acid, ethyl ester	0.48 (±0.15)	0.53 (±0.22)
Octanoic acid, propyl ester	0.48 (±0.08)	0.52 (±0.12)
Total esters	3.88	3.95
Acetic acid *	28.35 (±1.66)	23.75 (±1.15)
Propanoic acid *	6.94 (±0.72)	5.61 (±0.48)
Butanoic acid	1.23 (±0.22)	1.08 (±0.31)
Butanoic acid, 3-methyl	2.96 (±0.18)	2.85 (±0.33)
Hexanoic acid *	1.16 (±0.26)	0.34 (±0.19)
Octanoic acid	2.14 (±0.36)	1.75 (±0.24)
n-Decanoic acid	1.57 (±0.15)	1.39 (±0.35)
n-Hexadecanoid acid *	14.30 (±0.97)	12.51 (±0.90)
Total acids	58.65	49.28

## References

[1] Tremonte P., Reale A., Coppola R., Succi M. (2005). Preliminary investigations on microbiological characteristics of “Ventricina”. Ind. Aliment..

[2] Tremonte P., Sorrentino E., Pannella G., Tipaldi L., Sturchio M., Masucci A., Maiuro L., Coppola R., Succi M. (2017). Detection of different microenvironments and *Lactobacillus sakei* biotypes in Ventricina, a traditional fermented sausage from central Italy. Int. J. Food Microbiol..

[3] Leroy F., Scholliers P., Amilien V. (2015). Elements of innovation and tradition in meat fermentation: Conflicts and synergies. Int. J. Food Microbiol..

[4] Cocconcelli P.S., Fontana C., Toldrá F. (2010). Starter cultures for meat fermentation. Handbook of Meat Processing.

[5] Leroy S., Lebert I., Talon R., Toldrá F., Hui Y.H., Astiasarán I., Sebranek J.G., Talon R. (2015). Microorganisms in Traditional Fermented Meats Dry-Fermented Sausages and Ripened Meats: An Overview. Handbook of Fermented Meat and Poultry.

[6] Callahan B.J., McMurdie P.J., Rosen M.J., Han A.W., Johnson A.J., Holmes S.P. (2016). DADA2: High-resolution sample inference from Illumina amplicon data. Nat. Methods.

[7] Davis N.M., Proctor D.M., Holmes S.P., Relman D.A., Callahan B.J. (2018). Simple statistical identification and removal of contaminant sequences in marker-gene and metagenomics data. Microbiome.

[8] Montanari C., Gatto V., Torriani S., Barbieri F., Bargossi E., Lanciotti R., Grazia L., Magnani R., Tabanelli G., Gardini F. (2018). Effects of the diameter on physico-chemical, microbiological and volatile profile in dry fermented sausages produced with two different starter cultures. Food Biosci..

[9] Tabanelli G., Pasini F., Riciputi Y., Vannini L., Gozzi G., Balestra F., Caboni M.F., Gardini F., Montanari C. (2018). Fermented nut-based vegan food: Characterization of a home-made product and scale-up to an industrial pilot-scale production. J. Food Sci..

[10] Pasini F., Soglia F., Petracci M., Caboni M.F., Marziali S., Montanari C., Gardini F., Grazia L., Tabanelli G. (2018). Effect of fermentation with different lactic acid bacteria starter cultures on biogenic amine content and ripening patterns in dry fermented sausages. Nutrients.

[11] Ferreira M.T., Manso A.S., Gaspar P., Pinho M.G., Neves A.R. (2013). Effect of oxygen on glucose metabolism: Utilization of lactate in *Staphylococcus aureus* as revealed by in vivo NMR studies. PLoS ONE.

[12] Zheng J., Wittouck S., Salvetti E., Franz C.M.A.P., Harris H.M.M., Mattarelli P., O’Toole P.W., Pot B., Vandamme P., Walter J. (2020). A taxonomic note on the genus *Lactobacillus*: Description of 23 novel genera, emended description of the genus *Lactobacillus* Beijerinck 1901, and union of *Lactobacillaceae* and *Leuconostocaceae*. Int. J. Syst. Evol. Microbiol..

[13] Aquilanti L., Garofalo C., Osimani A., Clementi F. (2016). Ecology of lactic acid bacteria and coagulase negative cocci in fermented dry sausages manufactured in Italy and other Mediterranean countries: An overview. Int. Food Res. J..

[14] Vidal-Carou M.C., Veciana-Nogués M.T., Latorre-Moratalla M.L., Bover-Cid S., Toldrá F., Hui Y.H., Astiasarán I., Sebranek J.G., Talon R. (2015). Biogenic amines: Risks and control. Handbook of Fermented Meat and Poultry.

[15] Comi G., Urso R., Iacumin L., Rantsiou K., Cattaneo P., Cantoni C. (2005). Characterisation of naturally fermented sausages produced in the northeast of Italy. Meat Sci..

[16] Aymerich T., Martin B., Garriga M., Vidal-Carou M.C., Bover-Cid S., Hugas M. (2006). Safety properties and molecular strain typing of lactic acid bacteria from slightly fermented sausages. J. Appl. Microbiol..

[17] Treviño E., Beil D., Steinhart H. (1997). Formation of biogenic amines during the maturity process of raw meat products, for example of cervelat sausage. Food Chem..

[18] European Commission (2005). Commission Regulation (EC) No. 2073/2005 of 15 November 2005 on microbiological criteria for foodstuffs. Off. J. Eur. Union L.

[19] Montanari C., Barbieri F., Magnani M., Grazia L., Gardini F., Tabanelli G. (2018). Phenotypic diversity of *Lactobacillus sakei* strains. Front. Microbiol..

[20] Rimaux T., Riviére A., Illeghems K., Weckx S., De Vuyst L., Leroy F. (2012). Expression of the arginine deiminase pathway genes in *Lactobacillus sakei* is strain-dependent and is affected by environmental pH. Appl. Environ. Microbiol..

[21] Marcobal A., de Las Rivas B., Landete J.M., Tabera L., Muñoz R. (2012). Tyramine and phenylethylamine biosynthesis by food bacteria. Crit. Rev. Food Sci. Nutr..

[22] Barbieri F., Montanari C., Gardini F., Tabanelli G. (2019). Biogenic amine production by lactic acid bacteria: A review. Foods.

[23] Suzzi G., Gardini F. (2003). Biogenic amines in dry fermented sausages: A review. Int. J. Food Microbiol..

[24] Alan Y., Topalcengiz Z., Dığrak M. (2018). Biogenic amine and fermentation metabolite production assessments of *Lactobacillus plantarum* isolates for naturally fermented pickles. LWT.

[25] Lee J.H., Jin Y.H., Park Y.K., Yun S.J., Mah J.H. (2019). Formation of biogenic amines in Pa (*Green onion*) Kimchi and Gat (*Mustard leaf*) Kimchi. Foods.

[26] Gardini F., Özogul Y., Suzzi G., Tabanelli G., Özogul F. (2016). Technological factors affecting biogenic amine content in foods: A review. Front. Microbiol..

[27] Rimaux T., Vrancken G., Vuylsteke B., De Vuyst L., Leroy F. (2011). The pentose moiety of adenosine and inosine is an important energy source for the fermented-meat starter culture *Lactobacillus sakei* CTC 494. Appl. Environ. Microbiol..

[28] Tabanelli G., Coloretti F., Chiavari C., Grazia L., Lanciotti R., Gardini F. (2012). Effects of starter cultures and fermentation climate on the properties of two types of typical Italian dry fermented sausages produced under industrial conditions. Food Control.

[29] Montanari C., Bargossi E., Gardini A., Lanciotti R., Magnani R., Gardini F., Tabanelli G. (2016). Correlation between volatile profiles of Italian fermented sausages and their size and starter culture. Food Chem..

[30] von Wright A., Axelsson L., Lahtinen S., Ouwehand A.C., Salminen S., von Wright A. (2011). Lactic acid bacteria: An introduction. Lactic Acid Bacteria: Microbiological and Functional Aspects.

[31] Ordóňez J.A., Hierro E.M., Bruna J.M., de la Hoz L. (1999). Changes in the components of dry fermented sausages during ripening. Crit. Rev. Food Sci. Nutr..

[32] Gänzle M.G. (2015). Lactic metabolism revisited: Metabolism of lactic acid bacteria in food fermentations and food spoilage. Curr. Opin. Food Sci..

[33] Perea-Sanz L., López-Díez J.J., Belloch C., Flores M. (2020). Counteracting the effect of reducing nitrate/nitrite levels on dry fermented sausage aroma by *Debaryomyces hansenii* inoculation. Meat Sci..

[34] Lorenzo J.M., Gómez M., Purriños L., Fonseca S. (2016). Effect of commercial starter cultures on volatile compound profile and sensory characteristics of dry-cured foal sausage. J. Sci. Food Agric..

[35] Olivares A., Dryahina K., Navarro J.L., Smith D., Spanĕl P., Flores M. (2011). SPME-GC-MS versus selected ion flow tube mass spectrometry (SIFT-MS) analyses for the study of volatile compound generation and oxidation status during dry fermented sausage processing. J. Agric. Food Chem..

[36] Speranza G., Corti S., Fontana G., Manitto P. (1997). Conversion of meso-2,3-butanediol into 2-butanol by *Lactobacilli*. Stereochemical and enzymatic aspects. J. Agric. Food Chem..

[37] Flores M., Olivares A., Toldrá F., Hui Y.H., Astiasarán I., Sebranek J.G., Talon R. (2015). Flavor. Handbook of Fermented Meat and Poultry.

[38] Sánchez-Mainar M., Stavropoulou D.A., Leroy F. (2017). Exploring the metabolic heterogeneity of coagulase-negative staphylococci to improve the quality and safety of fermented meats: A review. Int. J. Food Microbiol..

[39] Smit B.A., Engels W.J.M., Wouters J.T.M., Smit G. (2004). Diversity of L-leucine catabolism in various microorganisms involved in dairy fermentations, and identification of the rate-controlling step in the formation of the potent flavor component 3-methylbutanal. Appl. Microbiol. Biotechnol..

[40] Gianelli M.P., Olivares A., Flores M. (2011). Key aroma components of a dry-cured sausage with high fat content (*Sobrassada*). Food Sci. Technol. Int..

[41] Olivares A., Navarro J.L., Flores M. (2009). Establishment of the contribution of volatile compounds to the aroma of fermented sausages at different stages of processing and storage. Food Chem..

[42] Carballo J., Mehta B.M., Kamal-Eldin A., Iwanski R.Z. (2012). The role of fermentation reactions in the generation of flavor and aroma of foods. Fermentation: Effects on Food Properties.

[43] Larrouture-Thiveyrat C., Montel M.C. (2003). Effects of environmental factors on leucine catabolism by *Carnobacterium piscicola*. Int. J. Food Microbiol..

[44] Larrouture-Thiveyrat C., Ardaillon V., Pepin M., Montel M.C. (2000). Ability of meat starter culture to catabolize leucine and evaluation of the degradation products by using an HPLC method. Food Microbiol..

[45] Masson F., Hinrichsen L., Talon R., Montel M.C. (1999). Factors influencing leucine catabolism by a strain of *Staphylococcus carnosus*. Int. J. Food Microbiol..

[46] Coll J., Leal J.A. (1972). Utilization of L-leucine as nitrogen source by fungi. Trans. Brit. Mycol. Soc..

[47] Stahnke L.H. (1995). Dried sausages fermented with *Staphylococcus xylosus* at different temperatures and with different ingredient levels—Part II. Volatile components. Meat Sci..

[48] Stavropoulou D.A., Borremans W., De Vuyst L., De Smet S., Leroy F. (2015). Amino acid conversions by coagulase-negative staphylococci in a rich medium: Assessment of inter- and intraspecies heterogeneity. Int. J. Food Microbiol..

